# Concentration of Penicillin G in Jawbone Affected by Antiresorptive Agent-Related Osteonecrosis Following a Single Preoperative Dose

**DOI:** 10.3390/antibiotics10010017

**Published:** 2020-12-27

**Authors:** Philipp Poxleitner, Michael Andreas Ermer, Rainer Trittler, Carolin Lena Feuerstein, Jörg-Elard Otten, Rainer Schmelzeisen, Pit Jacob Voss, David Steybe

**Affiliations:** 1 Medical Center, Department of Oral and Maxillofacial Surgery, Faculty of Medicine, University of Freiburg, 79106 Freiburg, Germany; philipp.poxleitner@uniklinik-freiburg.de (P.P.); caro-feuerstein@web.de (C.L.F.); joerg.elard.otten@uniklinik-freiburg.de (J.-E.O.); rainer.schmelzeisen@uniklinik-freiburg.de (R.S.); pit.voss@uniklinik-freiburg.de (P.J.V.); david.steybe@uniklinik-freiburg.de (D.S.); 2 Berta-Ottenstein-Programme for Clinician Scientists, Faculty of Medicine, University of Freiburg, 79106 Freiburg, Germany; 3 Pharmacy, Medical Center, Faculty of Medicine, University of Freiburg, 79106 Freiburg, Germany; rainer.trittler@uniklinik-freiburg.de

**Keywords:** antiresorptive agent-related osteonecrosis of the jaw, treatment, antimicrobial prophylaxis, penicillin, high performance liquid chromatography with tandem mass spectrometry (HPLC-MS/MS)

## Abstract

The aim of this study was to evaluate the concentration of penicillin G in bone affected by antiresorptive agent-related osteonecrosis of the jaw (ARONJ) following a single preoperative dose of 10 million international units (6000 mg). ARONJ is a major concern in patients administered antiresorptive agents for conditions associated with pathologically increased bone resorption. Antibiotic therapy is a key component of most treatment approaches for ARONJ and penicillin based regimens, providing a cost effective therapy option with a favorable side effect profile, are administered most frequently. In this study, high performance liquid chromatography with tandem mass spectrometry (HPLC-MS/MS) was applied to evaluate penicillin G concentration in serum and bone samples of 19 patients suffering from ARONJ and undergoing surgical treatment under perioperative intravenous (IV) antibiotic therapy. Penicillin G bone concentrations were above the limit of detection (0.1 μg/g bone tissue) in 16 out of 19 samples, with a median concentration of 2.7 μg/g (range 0.1–8.8 μg/g). Penicillin G concentrations in intraoperative serum samples were above the limit of detection in all serum samples, with a median concentration of 116 μg/mL (range 1–232 μg/mL). Thus, considering bacteria frequently found in ARONJ lesions, penicillin G at levels providing adequate antimicrobial activity was detected in the serum and 16 out of 19 osteonecrotic lesions of patients suffering from ARONJ.

## 1. Introduction

Antiresorptive agents like bisphosphonates or denosumab are a major component of the medical treatment of conditions associated with pathologically increased bone resorption (e.g., skeletal-related events in cancer patients or osteoporosis). Antiresorptive agent-related osteonecrosis of the jaw (ARONJ) is a major concern in these patients [1]. Despite extensive research conducted into this condition since it has first been reported in 2003, to date there is no universally accepted hypothesis on the pathophysiology of ARONJ. However, besides bone remodeling inhibition and invasive dentoalveolar surgical procedures, local microbial colonization and consecutive inflammation is considered a key factor in its pathogenesis [2]. This assumption is supported by clinical data, in which *Actinomyces*, *Fusobacteria*, *Streptococcus*, *Staphylococcus*, and *Treponema* species are reported as a frequent feature of ARONJ [3,4,5].

As of now, there is no consensus on the treatment of ARONJ, with various treatment strategies having been proposed that can generally be grouped into conservative/non-surgical and surgical approaches [6]. Despite the heterogeneity within the treatment strategies proposed, with infection considered an important factor in the pathogenesis and progression of ARONJ, antibiotic therapy can be found as a component of most treatment approaches [1,7,8,9].

Conservative approaches generally aim at eliminating pain, controlling infection, and minimizing the progression of bone necrosis [10]. In this context, antibiotics are considered an important measure to control infection of hard and soft tissue [8,11]. Whereas conservative approaches mainly aim at controlling the disease, surgical treatment approaches for ARONJ aim at definite elimination of osteonecrosis and mucosal closure at the site of disease [12]. With various investigators having reported high success rates applying a perioperative antibiotic regimen in surgical treatment for ARONJ, this measure has become an essential part of the surgical treatment approach. Regarding this aspect, the current ARONJ guideline by the German Dental and German Oral and Maxillofacial Associations states the administration of systemic antibiotics intended as an adjunct to surgery to be obligatory in all operative ARONJ treatment. However, no specific recommendations are made with respect to the duration or route of administration [9]. 

As with the treatment approaches for ARONJ in general, heterogeneity can be found in the literature regarding the antibiotic regimens administered. However, a recent literature review of perioperative antibiotic administration in surgery for MRONJ/ARONJ found that penicillin-based antibiotics (alone or in combination with beta-lactamase inhibitors or metronidazole) were the agents administered most frequently [13]. 

Treatment of bone infections is challenging as the efficacy of antibiotic treatment depends significantly on the penetration capacity of the administered antibiotic to the infection site [14]. In bone tissue, this penetration capacity is lower than in other, well-perfused tissues and can be further impaired in the case of poorly vascularized or necrotic bone [15].

In the past, studies have been conducted to analyze the antibiotic concentration in (healthy) jawbone after intravenous administration of penicillin G [16], piperacillin/tazobactam [17], cefuroxime [18], and clindamycin [19]. In all of these studies, the authors concluded that a single intravenous dose of the antibiotic agent investigated could provide adequate antimicrobial prophylaxis [16,17,18,19]. Evaluating the concentration of intravenously administered ampicillin/sulbactam in irradiated jawbone, Heibel et al. [20] found concentrations 3–4 times lower than in healthy, non-irradiated bone. However, these concentrations were still above the minimum inhibitory concentration (MIC) of frequent oral pathogens [20].

To the authors’ knowledge, to date, there are no data available on the concentration of antibiotics achievable in bone affected by ARONJ. Thus, the aim of this study was to obtain qualitative and quantitative data on penicillin G concentration in bone affected by ARONJ following intravenous (IV) administration.

## 2. Results

The patients included in this study had a median age of 72.5 years (range 51–81 years) and a median BMI of 23.4; four of the patients were smokers. Fifteen patients were administered antiresorptive therapy for malignant diseases and four patients were administered antiresorptive therapy for osteoporosis. Twelve patients were administered bisphosphonates (11 patients zoledronic acid, one patient pamidronic acid), six patients were administered denosumab, and one patient had been administered bisphosphonates and denosumab. Twelve patients presented with ARONJ in the mandible, six patients presented with ARONJ in the maxilla, and one patient presented with ARONJ in both jaws. Table 1 summarizes the data on the weight of the bone samples, the duration of the infusion of penicillin G, the interval between the start of infusion and bone resection as well as on the concentration of penicillin G in the bone and serum samples.

The bone samples had a median weight of 76 mg (range 16–550 mg). The median interval between the start of infusion and the resection of bone was 43 min (range 29–88 min). Concentrations of penicillin G were above the limit of detection in 16 out of 19 bone samples; the median penicillin G concentration in bone was 2.7 μg/g (range 0.1–8.8 μg/g).

The median duration of penicillin G infusion was 11 min (range 6–22 min). Penicillin G concentrations were above the limit of detection in all intraoperative serum samples; the median concentration of penicillin G in the intraoperative serum samples was 116 μg/mL (range 1–232 μg/mL). No penicillin G was detected in the preoperative serum samples. There was a ratio of 1:43 between the median bone and serum concentration of penicillin G. 

## 3. Discussion

The aim of this study was to quantify the level of penicillin G concentration achievable in jawbone affected by antiresorptive-agent related osteonecrosis by measuring penicillin G jawbone concentrations following a single preoperative dose of 10 million IU (6000 mg) administered to patients with a baseline penicillin G serum concentration < LOD.

Penicillin G was detected in all bone samples except for three, in which concentrations were below the limit of detection of 0.1 μg/g (Table 1). Considering the weight of the bone samples, it can be noticed that all bone samples with penicillin concentrations below the limit of detection were relatively large and that high concentrations of penicillin G were mostly found in smaller bone samples. However, no statistically significant correlation could be found regarding this aspect (correlation coefficient: −0.208; p: 0.393). Necrotic bone is poorly vascularized and supplied mainly by the periosteum and surrounding soft tissue; this might serve as an explanation for the concentrations of penicillin G being below the limit of detection in three samples. 

In our study, the median penicillin concentration in jawbone affected by ARONJ was 2.7 μg/g (2.3% of the serum concentration) in bone specimens obtained at 18 to 72 min after completion of infusion of 10 million IU (6000 mg) of penicillin G. As expected, this value was lower than the one reported for healthy jawbone: In an investigation on penicillin G concentration in patients undergoing wisdom teeth extraction, Otten et al. [16] reported the mean concentration of penicillin G to be 17.4 μg/g (4.8% of the serum concentration) in compact jawbone specimens obtained between 6 and 140 min after completion of an infusion of 10 million IU (6000 mg) of penicillin G. 

Evaluating the concentration of ampicillin in irradiated jawbone, Heibel et al. [20] found a concentration of ampicillin/sulbactam of 5.51/1.21 µg/g after two doses of 3 g of ampicillin/sulbactam in bone specimens obtained at 110–430 min after infusion of the first dose of ampicillin/sulbactam. In line with our results, this concentration was much lower than the ampicillin/sulbactam concentration reported for healthy bone [20].

In their study on penicillin G concentration in healthy jawbone, Otten et al. reported bone concentrations to be highest within the first 40 min after the end of infusion [16] and a comparable relation between time and bone concentration was also confirmed by investigations on penicillin concentrations in other bones [22,23]. Thus, we aimed to obtain bone samples within a narrow time frame of <1 h after the end of penicillin G infusion, which was possible in all but one patient.

To provide a basis for clinical conclusions from the bone and serum concentrations of penicillin G determined in our study, the literature was searched for bacteria frequently found in bone affected by ARONJ [3,4,5] and minimum inhibitory concentration (MIC/MIC90) values for these bacteria were added from respective reports (Table 2). Due to the specific weight of bone, our values for penicillin G bone concentration, given in µg/g, were multiplied by 1.5 according to Landsdorfer et al. [21], in order to compare them to the MIC values found in the literature, given in µg/mL. Comparing the penicillin G concentrations found in our study to the MIC values of bacteria commonly found in bone affected by ARONJ, concentrations above the MIC90 for *Streptococcus mutans*, *Streptococcus anginosus*, *Fusobacterium nucleatum*, *Parvimonas micra*, and *Eubacterium spp.* were found in 16 samples. Penicillin G concentrations above the MIC90 for *Streptococcus sanguinis*, *Lactobacillus spp*., and *Actinomyces spp*. were found in 14 samples and penicillin G concentrations above the MIC90 for *Prevotella intermedia* were found for six samples. Penicillin G at bacteriostatic or even bactericidal concentrations regarding the pathogens listed in Table 2 was detected in 18 out of 19 serum samples (Table 1). One serum sample with an implausibly low value of 1 µg/mL was classified as the identification error, after a control measurement resulted in the same value. Contamination of the sample with saline solution at the time of blood draw or contamination during subsequent processing might be possible explanations for this finding. 

Whereas invasive surgical approaches aiming at complete elimination of necrotic bone yielded high success rates, these were much lower in approaches performing (superficial) debridement only [12,24]. Regarding conservative treatment of ARONJ foci, there is evidence from the literature indicating that the burden of necrotic bone commonly increases in these cases over time [12]. In this context, it has to be considered that necrotic bone will interfere with wound healing and mucosal recovery as dead tissue cannot be resurrected but will rather continue to exist as a complex chronic source of infection when left untreated [7,12]. Our results suggest that, especially in the case of larger necrotic areas and regarding obligate anaerobic bacteria like *Prevotella intermedia*, even high dose intravenous administration of penicillin G may not be sufficient to achieve an adequate antimicrobial effect in bone affected by ARONJ. Thus, in conservative treatment approaches, a beneficial effect of the (long-term) administration of antibiotic therapy seems questionable. 

In general, the results of our study can be considered to support the need for surgical resection in ARONJ treatment approaches with a curative intention. In ARONJ therapy approaches aiming at complete surgical resection of necrotic bone, perioperative administration of antibiotics has been reported as an important factor for successful treatment [25,26,27,28]. Analyzing the tissue surrounding ARONJ lesions, the bacteria observed in the center of the necrotic bone were also found in visually healthy bone adjacent to the necrotic bone [29] and in soft tissue associated with the necrotic bone [30]. Although not providing concentrations suitable for use as a conservative treatment measure, the levels of penicillin G achievable in bone affected by ARONJ might thus serve to pre- and intraoperatively reduce the bacterial burden resulting from the necrotic bone. Considering the serum penicillin G concentrations found in our study as well as the results of previous reports [16], it can moreover be assumed that the non-necrotic, normally vascularized jawbone adjacent to the necrotic tissue as well as the soft tissue can be provided with an adequate concentration of penicillin G to achieve adequate perioperative antimicrobial activity in patients undergoing surgical ARONJ treatment. Thus, a prolonged perioperative antibiotic therapy can be considered as an adequate adjunct measure to support the healing process in surgically treated ARONJ.

It can be seen as a shortcoming of this study that we did not consider patient specific parameters like smoking status, BMI, or the type of antiresorptive agent administered. These factors might have had an impact on the penicillin G concentrations found in our study; however, evaluating the pharmacodynamic impact of these parameters would require a larger sample size. Moreover, while only bone tissue determined to be necrotic by visual evaluation (yellowish-greenish, visually non-perfused) was analyzed, it cannot be fully ruled out that small amounts of healthy bone were unintentionally included in some of the analyzed samples. 

As penicillin-based regimens are the ones administered most frequently in the treatment of ARONJ [13], there is a rationale for an investigation into the penicillin G levels achievable in patients suffering from this condition. In our department, we are now administering a regimen consisting of intravenous penicillin G 10 million IU (6000 mg) twice a day two days before and five days after surgery, supplemented by intravenous metronidazole 500 mg twice a day. Levels of penicillin G above the MIC90 for a number of bacteria frequently found in ARONJ lesions could be demonstrated in this study in the serum and in 16 out of 19 osteonecrotic lesions. However, it has to be considered that obligate anaerobic bacteria (e.g. *Prevotella intermedia*) have relatively high MIC90 values for penicillin G. Thus, it might be advisable to apply a combined regimen with penicillin and metronidazole as we do at our center.

While the literature provides some information on the concentrations of antibiotics achievable in jawbone following IV administration, with this study adding data on concentrations achievable in jawbone affected by ARONJ, there is a lack of information on the concentration of antibiotics in jawbone following oral administration. As this is a route of administration commonly applied in clinical practice, a similar study might be carried out focusing on antibiotics administered orally.

## 4. Materials and Methods 

This non-randomized, non-blinded, prospective clinical investigation was performed at the Department of Oral and Maxillofacial Surgery, University Medical Center Freiburg, Germany after obtaining approval by the University of Freiburg Ethics Committee (Reference 287/13). All patients included in this study provided written informed consent.

Patients were included, if they

(i.) presented with a clinically and radiographically confirmed diagnosis of ARONJ;
(ii.) had an indication for surgical resection of necrotic bone with subsequent primary closure and preoperative/perioperative administration of IV antibiotic therapy (penicillin G 10 million IU and metronidazole 500 mg); and
(iii.) provided informed written consent for this study.

Patients were excluded if they

(i.) presented with osteonecrosis of the jaw due to other medical circumstances (e.g., irradiation);
(ii.) presented with an allergy to penicillin and/or metronidazole;
(iii.) were pregnant or breastfeeding; and
(iv.) were minor or incapacitated to provide informed consent.

A bone sample as well as pre- and intraoperative blood samples were obtained from a total of 19 patients meeting all inclusion criteria. Control samples were obtained from two patients that underwent procedures with the resection of alveolar bone and without an indication for the administration of antibiotics. 

As part of this study, the point in time of pre- and intraoperative blood draw, the point in time and duration of administration of preoperative IV antibiotic therapy and the point in time of bone resection were documented.

### 4.1. Preparation of Serum and Bone Samples

Venous blood was drawn prior to the IV administration of antibiotic therapy (penicillin G 10 million IU and metronidazole 500 mg) and directly after surgical resection of the necrotic bone using serum tubes (Sarstedt AG & Co, Nümbrecht, Germany). Immediately after blood draw, the serum tubes were taken to the laboratory, where the samples where centrifuged at 5300 rpm for 5 min. The serum obtained this way was pipetted into cryotubes and deep-frozen at −80 °C. For further processing, the serum samples were thawed, diluted in methanol in a 1:2 proportion, and subsequently centrifuged at 5300 rpm for 5 min. The supernatant was pipetted into screw thread vials and deep-frozen at −80 °C until HPLC-MS/MS analysis.

Bone samples were obtained from the bone affected by ARONJ directly after surgical removal. Subsequently, any adherent tissue was removed from the samples using sterile surgical instruments and all samples were cleaned using a moisturized sterile swab until they were macroscopically free of blood, as has been reported in the literature [16,18]. Rinsing of the samples was avoided to rule out the risk of the dilution of penicillin G within the bone. All bone samples were deep-frozen in liquid nitrogen at −80 °C immediately. Samples with a weight exceeding 100 mg (nine samples) were ground using a bone mill (Medicon eG, Tuttlingen, Germany). To avoid a loss of bone tissue, samples with a weight of less than 100 mg (10 samples) were processed using a tissuelyser (Qiagen GmbH, Hilden, Germany) at 30 Hz for 2 min. The bone powder resulting from both processing techniques was suspended in phosphate buffer solution (PBS) in a proportion of 1:10. This was followed by vortexing for 1 min, an ultrasonic bath, and centrifugation at 5300 rpm. Subsequently, the supernatant was pipetted into screw thread vials and deep-frozen at −80 °C for further processing.

### 4.2. Calibration Standards

Calibration standards were prepared from blank (penicillin G free) blood and bone of the control group, processed the same way as described above. Standard antibiotic solutions (100 mL; penicillin G plus PBS) were prepared using the following amounts of penicillin G: 10 mL, 5 mL, 1 mL, 500 μL, 100 μL, 50 μL, 25 μL, and 0 μL. To prepare the bone standards, 0.5 g of the antibiotic-free bone sample was mixed with 5 mL of each of the standard antibiotic solutions. Serum standards were prepared in the same manner.

### 4.3. Determination of IV Antibiotic Concentration in Bone and Serum

Concentrations of penicillin G in bone and serum were determined by the University Medical Center Freiburg Pharmacy by applying high performance liquid chromatography (Dionex) with quadrupole time-of-flight (q-TOF) mass spectrometry (Bruker, Bremen). Defined sample volumes of 2 μL (serum) and 20 μL (bone extract) were applied to the liquid chromatography (LC) column (Reposil-Pur Basic C18 100 × 2 mm, Maisch GmbH, Ammerbuch-Entringen, Germany). The calibration standards were used to obtain a calibration row as the basis for calculating the penicillin G concentrations. In this study, resulting from the method applied, the limit of detection (LOD) for penicillin G was 0.1 μg/g bone tissue. 

## 5. Conclusions

In this study, penicillin G at levels providing adequate antimicrobial activity/prophylaxis was found in the serum and 16 out of 19 osteonecrotic lesions of patients suffering from ARONJ. Thus, systemic (IV) administration of penicillin based antibiotic regimens can potentially serve as a useful adjuvant measure in surgical ARONJ treatment regimens aiming at complete elimination of necrotic bone. 

## Figures and Tables

**Table 1:** Summary of bone and serum concentration of penicillin G, weight of bone samples, infusion parameters of penicillin G, and body mass index (BMI) of the patient sample.

Sample Number	Extracted Penicillin G per g Bone (μg/g)	Bone Concentration Calculated (μg/mL) ^1^	Bone Weight (mg)	Serum Concentration Preoperative (μg/mL)	Serum Concentration Intraoperative (μg/mL)	BMI	Duration of Infusion	Intervals: Start of Infusion to Resection of Bone
1	<LOD	<LOD	536	<LOD	104	23.1	14	45
2	1.0	1.5	16	<LOD	43	23.5	9	32
3	2.1	3.15	41	<LOD	208	21.4	10	31
4	0.1	0.15	29	<LOD	118	23.6	16	88
5	2.0	3.0	155	<LOD	144	29.7	14	54
6	0.2	0.3	39	<LOD	37	32.5	11	58
7	5.7	8.55	20	<LOD	113	27.7	16	40
8	<LOD	<LOD	411	<LOD	116	20.8	15	80
9	1.3	1.95	550	<LOD	79	33.1	6	62
10	7.3	10.95	176	<LOD	171	20.2	9	36
11	2.8	4.2	107	<LOD	119	16.9	6	29
12	5.3	7.95	76	<LOD	193	24.2	8	37
13	2.5	3.75	116	<LOD	116	24.8	14	43
14	4.3	6.45	26	<LOD	232	23.2	6	58
15	3.1	4.65	26	<LOD	203	19.1	8	30
16	<LOD	<LOD	118	<LOD	46	23.0	22	40
17	3.9	5.85	76	<LOD	131	21.8	18	61
18	8.8	13.2	215	<LOD	1	23	10	33
19	1.9	2.85	69	<LOD	103	27.5	12	48
**median**	**2.7**	**3.98**	**76**		**116**	**23.2**	**11**	**43**

LOD: limit of detection, ^1^ Calculated by multiplying the bone concentration (in µg/g) by 1.5 [21].

**Table 2:** Bacteria frequently found in antiresorptive agent-related osteonecrosis of the jaw (ARONJ) lesions and respective minimum inhibitory concentration (MIC/MIC90) for penicillin G.

Bacteria Frequently Found in ARONJ Lesions ^1^	MIC Range Penicillin G (μg/mL)	MIC90 Penicillin G (μg/mL)	Author
*Streptococcus anginosus group*	<0.06–0.25	<0.06	Obszanska et al. [31]
*Streptococcus mutans*	0.016–0.031	0.031	Järvinen et al. [32]
*Streptococcus sanguinis*	<0.3–1	0.5	Teng et al. [33]
*Lactobacillus spp.*	0.015–1	0.5	Citron et al. [34]
*Actinomyces spp.*	0.06–1	0.5	Lerner et al. [35]
*Prevotella intermedia*	<0.001–33	5	van Winkelhoff et al. [36]
*Fusobacterium nucleatum*	0.016–1	0.064	Jacinto et al. [37]
*Parvimonas micra*	0.016	0.016	van Winkelhoff et al. [36]
*Eubacterium spp.*	<0.03–0.5	0.125	Merriam et al. [38]

^1^ According to Pushalkar et al. [30].

## Data Availability

The data presented in this study are available on request from the corresponding author. The data are not publicly available due to privacy reasons.
